# It’s not “all in your head”: critical knowledge gaps on internalized HIV stigma and a call for integrating social and structural conceptualizations

**DOI:** 10.1186/s12879-019-3704-1

**Published:** 2019-03-05

**Authors:** Marija Pantelic, Laurel Sprague, Anne L. Stangl

**Affiliations:** 1 Frontline AIDS, Secretariat, Preece House, 91-101 Davigdor Rd, Brighton, Hove, BN3 1RE UK; 2 Department of Social Policy and Intervention, University of Oxford, Barnett House, 32 -37 Wellington Square, Oxford, OX1 2ER UK; 3 Global Network of People Living with HIV (GNP+), Eerste Helmersstraat 17 B3 I, 1054 CX Amsterdam, The Netherlands; 4 Department of Global Health, Youth and Development, The International Center for Research on Women, 1120 20th St. NW Suite 500N, Washington, DC 20036 USA

**Keywords:** HIV, AIDS, Stigma, Internalized stigma, Theory, Ethics, Post-individualistic

## Abstract

**Background:**

Internalized HIV stigma is a public health concern as it can compromise HIV prevention, care and treatment. This paper has two aims. First, it highlights the urgent need for research evidence on internalized HIV stigma based on critical knowledge gaps. Here, critical knowledge gaps were identified based on most up-to-date systematic review-level evidence on internalized stigma related to HIV and mental health difficulties. Secondly, the paper calls for a shift in focus of internalized HIV stigma research, one that moves beyond psychological frameworks to integrate social, structural and intersectional conceptualizations of stigma. This part of the paper reviews the evolution of stigma theory since Goffman’s 1963 seminal work - which defined stigma - to present.

**Main text:**

Despite studies consistently suggesting that internalized HIV stigma is more prevalent than enacted stigma, there is little evidence of well-established programs to address it. In addition to this, considerable gaps in basic knowledge about the drivers of internalized HIV stigma hamper the development of an evidence-based response to the problem. The limited intervention and epidemiological research on the topic treats internalized HIV stigma as a purely psychological phenomenon. The second part of the paper provides arguments for studying internalized HIV stigma as a function of social and structural forces: (1) Individual-level interventions for internalized HIV stigma are rooted in out-dated theoretical assumptions; (2) From an ethics point of view, it could be argued that individual-level interventions rely on a victim-centric approach to a public health problem; (3) Social and structural approaches to internalized HIV stigma must be explored due to the high opportunity cost associated with small-scale individual-level interventions.

**Conclusions:**

Critical gaps in intervention and epidemiological research in internalized HIV stigma remain. There has been an absence of a shared, sound theoretical understanding of internalized HIV stigma as a manifestation of social and structural factors. This commentary sought to stimulate a dialogue to remedy this absence. Future research should take into account ethical considerations, the evolution of stigma theory over the past five decades, intersectionality and opportunity cost when framing hypotheses, developing theories of change and designing interventions.

## Background

*Internalized HIV stigma* occurs when a person living with HIV endorses negative attitudes associated with HIV and accepts them as applicable to him or herself [32, 33]. It is characterized by feelings of shame, guilt and worthlessness [44, 77]. In addition to compromising the quality of life of people living with HIV, internalized HIV stigma can also have serious epidemiological consequences. Namely, internalized HIV stigma can contribute to the spread of sexually transmitted infections and HIV by instilling fear of rejection from sexual partners and low self-confidence [6], hampering condom use [34] and compromising adherence to anti-retroviral treatment [43].

Internalized HIV stigma may develop independently of discrimination [32, 33]. For example, when a person is diagnosed with HIV, she or he might decide not to disclose their status to others due to anticipated stigma [28]. This situation would make one susceptible to internalized HIV stigma but less so to overt HIV-related discrimination [26, 41, 59].

However, a growing body of research suggests that irrespective of whether one directly experiences HIV-specific discrimination, internalized HIV stigma is driven by broader social and structural determinants of health [17, 62, 73–75]. Social and structural determinants of health refer to the complex and often overlapping social structures, norms and practices, as well as economic and political disparities that have the power to shape and, at times, limit the health and wellbeing of individuals [23]. This theoretical perspective is rooted in the basic understanding that the psychology of individuals are influenced by their external environment, including their social networks, structures, and institutions [12].

For example, availability of antiretroviral treatment and economic structures can influence ‘instrumental and symbolic associations between HIV and premature morbidity, economic incapacity and death’ [76]. Social and structural violence against people living with HIV fuel anticipation of stigma [1, 17, 62, 74, 75, 79] such that, in the example above, the newly diagnosed person living with HIV would have developed perceptions about HIV-related stigma prior to their own diagnosis [45, 46]. Therefore although internalized HIV stigma may occur without having personally (or individually) experienced HIV-related discrimination, evidence strongly suggests that it is intimately linked with and shaped by social and structural forces.

This paper has two aims. First, it highlights the urgent need for research evidence on internalized HIV stigma based on critical knowledge gaps. Here, critical knowledge gaps were identified based on most up-to-date systematic review-level evidence on internalized stigma related to HIV and mental health difficulties. Secondly, the paper calls for a shift in focus of internalized HIV stigma research, one that moves beyond psychological frameworks to integrate social and structural conceptualizations of stigma. This part of the paper reviews the evolution of stigma theory since Goffman’s 1963 seminal work - which defined stigma - to present.

## Main text

### Critical knowledge gaps in internalized HIV stigma research

Existing intervention studies heavily focus on reducing enacted stigma [72], which refers to negative public attitudes or discrimination towards people living with HIV [42]. Despite studies from Argentina, Burkina Faso, Cambodia, Kenya, Russia, South Africa, consistently suggesting that internalized HIV stigma is more prevalent than enacted stigma [26, 58, 63, 66, 67, 84], there is limited evidence of well-established programs to address internalized HIV stigma [16, 72, 80, 81].

Moreover, considerable gaps in basic knowledge about the drivers of internalized HIV stigma hamper the development of an evidence-based response to the problem. A recent systematic review of internalized HIV stigma predictors in sub-Saharan Africa found few longitudinal studies and, within these, only individual-level predictors were assessed: poor HIV-related health and poor mental health were found to precede and drive increases in internalized HIV stigma over time [64]. However it could also be argued that internalized HIV stigma increases psychological distress [32, 33] and compromises HIV-related health through reduced adherence to ART [43]. The relationships between these risks are likely to be cyclical rather than linear but the vast majority of studies on this are cross-sectional, limiting inferences about order of effects [43, 64].

Despite the well-documented effects of social and structural risks on physical and mental health outcomes [20, 40, 55, 60, 61], internalized HIV stigma largely continues to be viewed as occurring in a cognitive and psychological vacuum. The majority of known interventions aiming to reduce internalized HIV stigma have focused on individual-level factors such as self-esteem and cognition among people living with HIV [72]. They have used small sample sizes, limiting inferences about effectiveness [72]. The literature on mental health-related internalized stigma also offers a number of personal empowerment, cognitive behavioural therapy and psycho-education interventions that aim to reduce internalized stigma [24]. However, a recent systematic review and meta-analysis of such interventions was unable to demonstrate their effectiveness in the long term in reducing internalized stigma [13]. In line with this, future intervention research must expand on existing psychological perspectives and take into account the social and structural forces that are likely to shape internalized HIV stigma [1, 15, 62, 65].

More research is also needed to better understand internalized stigma among caregivers of people living with HIV and its potential ripple effects on internalized stigma and health outcomes among people living with HIV. The broader mental health literature suggests that caregivers of people with mental health difficulties commonly experience internalized stigma, which may further affect their interactions with the patient [19, 37]. For example, caregivers experiencing internalized stigma may avoid being identified with the people that they are caring for [37], which may have serious implications for the quality of care and perceived and internalized stigma of the patient. Similarly, caregivers and family members of people living with HIV experience substantial amounts of stigma-by-association and the adverse effects of this on their mental health have been well documented [8–10, 22]. However, evidence is needed to better understand the effects of caregiver internalized stigma on people living with HIV; to disentangle internalized from other types of stigma-by-association; and to identify points for intervention.

### Towards social and structural conceptualizations of internalized HIV stigma

The theoretical, ethical and opportunity cost arguments for studying internalized HIV stigma as a function of social and structural environments are outlined below.

### Theory

Individual-level interventions for internalized HIV stigma are rooted in theoretical assumptions that arose after Goffman’s seminal work ‘Stigma: Notes on the Management of a Spoiled Identity’ [38]. Goffman defined stigma as a process through which individuals are ‘disqualified from full social acceptance’ due to an undesirable ‘mark’ or ‘label’*.* This label can be a physical, health or behavioural attribute that is deemed ‘deeply discrediting’. Such labels create the perception that the possessors have less desirable identities (or ‘spoiled identities’) than ‘normal’ people. Stigma, according to Goffman, reduces the possessor ‘from a whole and usual person to a tainted, discounted one’ [38]. Importantly, Goffman posited that stigma is rooted in social interactions. He highlighted that stigmatization requires more than mere labels; rather, a ‘language of relationships’ is essential. Hence, stigma consists of at least two essential components: (1) recognition of difference based on a mark or label and (2) consequent devaluation of the possessor of the mark [29].

However, in the years following Goffman’s seminal work, stigma theory became highly stigmatizing. The concept of stigma was applied to psychology, most prominently through Scheff’s ‘labelling theory of mental illness’. According to labelling theory, stigma was a product of the behavioural characteristics of both the labellers and the labelled [69, 70]. Here, labelling and symptoms of mental health difficulties were hypothesized to have a cyclical relationship. Scheff thought that whilst symptoms of mental health difficulties contributed to labelling of a person as having a particular disorder, labelling also affected the mental health and behaviour of individuals because the labelled conformed to the negative expectations. ‘When […] persons around the deviant react to him uniformly in terms of the stereotypes of insanity, his amorphous and unstructured rule-breaking tends to crystalize in conformity to these expectations, thus becoming similar to behaviour of other deviants classified as mentally ill’, states Scheff [69].

Early critics of labelling theory thought that symptomatic behaviour alone – and not labelling – contributed to stigma [18, 39]. In the late 70s and early 80s, such critics dominated the field [48]. They rejected the notion that labelling and poor mental health reinforce each other. For example, Gove believed that ‘the available evidence indicates that deviant labels are primarily a consequence of deviant behaviour and that deviant labels are *not* a prime cause of deviant careers’ (1975, emphasis added). Similar to labelling theory, its early critics placed a strong emphasis on the role of individual attributes in producing stigma. However, unlike proponents of labelling theory, they stressed that stigma was inconsequential. In other words, they denounced the potential outcomes of stigma, and considered stigma to be an outcome of personal traits and behavioural characteristics of people considered as ‘deviants’.

In response to these individualistic approaches to stigma, Link and colleagues constructed a modified labelling theory [45, 46, 48, 49]. They expanded on Goffman’s work and labelling theory, but rejected the notion that stigma was a direct product of the behavioural attributes of the stigmatized. According to modified labelling theory, stigma manifests itself ‘when elements of labelling, stereotyping, separation, status loss, and discrimination co-occur in a power situation that allows them to unfold’ [48]. As such, stigma is ‘highly situationally specific, dynamic, complex and nonpathological’ [29]. A key contribution of this post-individualistic approach is that it stresses that stigma occurs within social contexts characterized by power inequalities [47]. In 2003, Parker and Aggleton applied modified labelling theory to the study of HIV-related stigma. They defined HIV stigma as a process inherently linked to the maintenance of social and structural power inequalities. Parker and Aggleton highlighted the need to conceptualize HIV stigmas ‘as social processes that can only be understood in relation to broader notions of power and domination.’

More recent theoretical work has emphasized the importance of intersectional approaches for understanding the production of stigma in the context of HIV [7, 11, 30, 32, 33, 50, 53]. Intersectionality theory is grounded in the reality that people exist at a juncture of race, gender, class, sexual orientation and other identities, with a multiplicity of potential social positionings that reflect different distances from social power and regard based on these identities [25, 31]. In other words, the experience of living with HIV never occurs in a vacuum; the extent to which stigma is internalized may be alternately heightened or ameliorated based on other identities and how those identities are valued or devalued in a given community or society. An intersectional approach opens analytical space for voices that would be consigned even further to the margins when a positive HIV-status or any other identity is assumed to be universally experienced. In line with this, evidence suggests that internalized HIV-related stigma operates within mutually reinforcing relationships with other marginalized social statuses based on sex, age, gender identity and expression, racialisation, sexual orientation and behaviors, illicit drug or alcohol use, sex work, criminalization and incarceration [2]; [52, 36, 68]. A recent trial found that a financial savings promotion and psychological support intervention for sex workers in India resulted in significant reductions in internalized stigma related to sex work, as well as improvements in self-worth, health seeking behaviours and long-term savings [36]. However the adoption of the intersectionality framework for the study of internalised HIV-related stigma is still in nascent stages.

The questions an intersectional lens opens for exploration are rich: does belonging to a dominant group in any of these identities ameliorate or intensify the experience of internalized HIV stigma? Does lived experience responding to racism and sexism [51, 53], for example, provide translatable lessons for communities to maintain self worth and reject devaluing social messages associated with HIV? And if so, is this similar across sexual practices, drug use and other behaviours commonly associated with HIV? More empirical research is needed to better understand the most effective types of interventions to reduce the simultaneous effects of sexism, racism, ageism, ableism and other forms of ostracism on individuals’ wellbeing and internalized HIV stigma. Could, for example, social and structural interventions to increase social justice, such as for greater political voice or economic power, along one dimension of social hierarchies have positive spill over effects on internalized stigma across multiple dimensions? Gendered approaches that respond to specific needs of communities with intersecting vulnerabilities (e.g. women who inject drugs, transgender women sex workers, etc.) are also needed.

### Ethics

From an ethics point of view, it could be argued that individual-level interventions rely on a victim-centric approach to a public health problem [56]. Individual-level interventions situate the onus of change on the stigmatized. Such interventions may be able to reduce internalized HIV stigma at the individual level [78, 83], but they are not equipped to affect its sources at the social level [1, 15, 62, 79]. Hence, ‘the burden of adjustment falls on stigmatized individuals – with their responses conceptualized in terms of their individual abilities to adapt to the stress of stigma’ [14].

### Opportunity cost

Post-individualistic approaches to internalized HIV stigma must be explored due to the high opportunity cost associated with restricting programming and research to small-scale individual-level interventions. HIV epidemiology has already shifted from emergency HIV prevention that centred around individuals to more long-term, comprehensive, and strategic programming, also known as ‘combination prevention’ [5]. Recent reductions in funding to combat the HIV epidemic provide additional impetus to implement interventions that simultaneously address multiple needs and can be rolled out on a large scale. Some structural interventions, such as those aiming to tackle poverty and food insecurity, have the potential to simultaneously reduce internalized HIV stigma, avert new HIV and sexually transmitted infections and uphold the human rights of populations disproportionately affected by HIV [21, 27, 54, 57, 62, 73–76]. But the implementation of socio-structural interventions is a lengthy, painstaking process, often involving struggle, consensus building, and conflict resolution. Such interventions should complement, rather than replace, the shorter-term small-scale interventions against internalized HIV stigma, which can provide important support for people living with HIV and produce more immediate positive outcomes [78, 83].

At the same time, adverse social and structural forces continue to impede both the delivery and the effectiveness of existing individual-level interventions. In this respect, we have a lot to learn from HIV prevention efforts over the past three decades. Efficacious HIV prevention tools such as condoms, lubricants, and provision of sterile injecting equipment have existed since the onset of the epidemic. Prevention of vertical transmision (PVT) through ART was shown to be possible in 1994. Yet, the scale up of these evidence-based strategies has often been hampered by social and structural determinants: inequality, discrimination and punitive policies have continued to compromise HIV prevention, treatment and care. As a result, while progress has been made globally at slowing the epidemic’s progression, HIV infections continue to rise particularly among the most marginalized populations in under-resourced settings, including young women, sex workers, transgender people, people who use drugs, men who have sex with men, and migrants [82]. Despite medical advances, the end of AIDS will not be feasible without addressing the structural and social obstacles faced by communities most affected by HIV, who also bear the brunt of stigma and discrimination [3, 4, 71]. Similar stagnation in outcomes can be expected for internalized stigma interventions that disregard the broader socio-structural context.

Emerging evidence suggests that socio-structural interventions that successfully challenge HIV transmission risk and AIDS progression also hold promise for combatting internalized HIV stigma. For example, a prospective cohort study in Uganda found that access to anti-retroviral treatment reduced internalized HIV stigma over time [74, 75]. Another prospective impact evaluation of a 12-month food assistance intervention among 904 patients living with HIV in Uganda found that the program substantially reduced internalized stigma [54]. Qualitative data from Kenya also suggest that access to a livelihood intervention increased people’s confidence, self-esteem and productivity, and reduced feelings of HIV-related shame [76].

Including measures of internalised stigma as an additional outcome measure in other relevant socio-structural intervention studies could empirically test whether acting on social and structural determinants is associated with reductions in internalised stigma.

But without a precedent, it is unclear how interventions that are administered at the national or community level for people who are both HIV-positive and negative would measure their effects on internalised HIV stigma. Careful consideration of people’s privacy and confidentiality is warranted in the design and implementation of such evaluations. An HIV stigma measurement recently developed with and for adolescents living with HIV in South Africa offers two options – one with HIV-specific wording used for adolescents who self-disclose their HIV-positive status to the interviewer, and another with general mentions of health issues rather than HIV for status-unknown adolescents [63]. Tools such as this one may help researchers study HIV stigma in non-clinical settings whilst avoiding inadvertent disclosure of people’s HIV status.

## Conclusions

There has been an absence of a shared, sound theoretical understanding of internalized HIV stigma as a manifestation of social and structural factors. This commentary sought to stimulate a dialogue to remedy this absence. More than a decade ago, Parker and Aggleton [65] noted that a major limitation of studies on HIV-related stigma is that they fail to embed hypotheses or analyses within theoretical frameworks. Our analysis suggests that theoretically grounded intervention and epidemiological research on internalized HIV stigma is urgently needed. The reasons for why theoretical frameworks have largely gone missing from much research on HIV stigma are not known. However this situation might have arisen from the urgency researchers may feel to rapidly respond with interventions to address a social justice issue that effectively denies HIV care for marginalized groups of people. Unfortunately, as a result, critical gaps in basic knowledge around internalized HIV stigma and its manifestations and effects across groups of people living with HIV remain. The high prevalence of internalized HIV stigma and its epidemiological consequences warrant that these gaps be addressed. Future research should take into account ethical considerations, the evolution of stigma theory over the past five decades, intersectionality and opportunity cost when framing hypotheses, developing theories of change, and designing and evaluating interventions.

Social and structural interventions may help reduce internalised HIV stigma, but caution is warranted in such endeavours, as social and structural determinants are not static. Like stigma itself, they evolve over time and are culturally embedded. Therefore the relationship between broader social and structural determinants of HIV risk and internalized stigma may not be linear. Further, social and structural interventions arise from and exist within current interlocking systems of subordination and are subject to pressures to maintain existing power hierarchies. As a result, rigorous attention should be paid to ways in which interventions to reduce HIV vulnerability and improve HIV-related quality of life might simultaneously increase subordination of other identities. Longitudinal research is needed to unpack these complex relationships, evaluate long-term (and potentially harmful) outcomes of structural interventions and further advance theory.

It is essential to note that this article does not discount the role of psychological factors in the production and maintenance of internalized HIV stigma. Rather, it highlights the need to expand on current psychological frameworks, and integrate knowledge on the broader, contextual underpinnings of stigma. Even if internalized HIV stigma is an ‘internal’ psychological phenomenon, there is an urgent need to study how environmental factors affect it and how they may impede the delivery of individual-level interventions. Mental health is public health. Together, the extensive literature on the social and structural predictors of mental health and emerging evidence on internalized HIV stigma [35, 62, 79] clearly indicate that it’s not ‘all in your head’.
